# Relationship Between Quality of Life and Academic Performance in a Sample of Colombian University Students

**DOI:** 10.1192/j.eurpsy.2024.1248

**Published:** 2024-08-27

**Authors:** M. Paredes Bermúdez, M. Bayona Velásquez, M. Lora Monsalve, J. Viloria Escobar, K. L. Perez Correa

**Affiliations:** Magdalena, Instituto Nacional de Formación Técnica Profesional Humberto Velásquez García, Cienaga; ^2^Magdalena, Universidad Cooperativa de Colombia, Santa Marta, Colombia

## Abstract

**Introduction:**

Quality of life encompasses a multidimensional component that includes aspects of lifestyle, health, housing, personal satisfactions, which can affect the academic performance of students in their university studies.

**Objectives:**

To determine the relationship between the quality of life and academic performance of students at the National Institute of Professional Technical Training “Humberto Velásquez García” in Ciénaga, Colombia.

**Methods:**

Cross-sectional observational study involving a sample of 344 undergraduate students who completed the WHOQOL-BREF questionnaire, a sociodemographic form, and were asked about their academic performance in the last semester. Data were analyzed using RStudio, where categorical variables were interpreted through relative and absolute frequencies, and quantitative variables through medians. Bivariate analysis was conducted using non-parametric tests such as Mann-Whitney U and Kruskal-Wallis for group comparisons, and Kendall for correlations.

**Results:**

Academic performance had a median of 4.00, and the quality of life had a median of 47.57. The Mann-Whitney U test showed p=0.03 for gender-based performance comparison. Kruskal-Wallis comparison by age group regarding performance showed p=0.003. The correlation between academic performance and quality of life showed tau=0.120 and p=0.004.

**Conclusions:**

The median academic performance is above the approval point, but the quality of life is below average levels (on a scale of 1 to 100). There are significant differences in median performance among gender and age groups, as well as a very low, positive, and statistically significant correlation between academic performance and levels of quality of life.

**Disclosure of Interest:**

None Declared

